# Effects of PCNA Stability on the Formation of Mutations

**DOI:** 10.3390/ijms25168646

**Published:** 2024-08-08

**Authors:** Matan Arbel-Groissman, Batia Liefshitz, Martin Kupiec

**Affiliations:** The Shmunis School of Biomedicine & Cancer Research, The George S. Wise Faculty of Life Sciences, Tel Aviv University, Ramat Aviv 69978, Israel

**Keywords:** *Saccharomyces cerevisiae*, yeast, mutagenesis, DNA replication, DNA repair, DNA Damage Tolerance, trans-lesion synthesis, damage avoidance

## Abstract

The fidelity of replication, especially in the presence of DNA damage, is essential for the proper function of cells. Mutations that inactivate genes involved in DNA damage repair or bypass are enriched in several types of cancer cells. Thus, it is important to further our understanding of the mechanisms governing replication fidelity. PCNA is a ring-shaped complex that encircles DNA at the front of the replication fork, at the double-stranded/single-stranded DNA junction. It serves as a processivity factor for the different DNA replication polymerases, allowing them to replicate longer stretches of DNA by physically tethering them to the DNA and preventing their detachment. In addition, PCNA also regulates and coordinates different DNA damage bypass pathways meant to allow DNA replication in the presence of DNA damage. Due to its essentiality and the numerous functions it has in the cell, much is still unclear about PCNA. Here, we utilize PCNA mutants that lower the stability of the PCNA complex on the chromatin, and thus tend to disassociate and fall from the DNA. Using these mutants, we show that PCNA’s physical presence on the DNA can prevent DNA misalignment at repetitive sequences, leading to increased mutation formation. We also show that PCNA-interacting proteins play an important role in strengthening the ring’s stability on the chromatin. Such repetitive sequence-induced mutations are common in several human diseases and it is important to study their formation and the mechanisms guarding against them.

## 1. Introduction

Since mutagenesis randomly affects protein-coding genes, most mutations are probably deleterious to the cell and, in humans, they may contribute to cancer development [1]. The replicative DNA polymerases, Polδ and Polε, possess a proofreading ability that dramatically lowers the mutation rate when functioning properly [2]. Mutations in genes encoding DNA polymerases appear to be enriched in certain cancer types, and exhibit a mutator phenotype that can act as a driver of cancer development [3]. 

DNA polymerases synthesize the new DNA strand by complementing the information present at the old one. DNA damage may render regions of the DNA unrecognizable by the DNA polymerase (e.g., nucleotides may be chemically modified, distorted, or fused to neighboring nucleotides). In that case, the DNA polymerase is unable to recognize them and simply stops and stalls [4]. This condition is harmful to the cell and can result in genomic instability and even cellular death [5]. For this reason, DNA Damage Tolerance (DDT) mechanisms have evolved, allowing the replication to continue in the presence of damaged DNA, bypassing the lesion, which can be repaired at a later time. There are several DDT mechanisms, some of which are error-free and do not create any mutations while bypassing the damaged region and some are mutagenic, leaving mutations in their wake [6]. 

PCNA is a homotrimeric sliding clamp complex composed of three subunits of the Pol30 protein, which serves as the processivity factor for the DNA polymerases while also functioning as the main regulator of the DDT pathways [7]. PCNA is highly conserved and essential and is involved in DNA replication, epigenetic silencing, DNA damage repair/bypass, telomere maintenance and other nuclear functions [8]. It has been previously shown that *pol30* mutants with decreased complex stability have increased mutation rates [9], especially ‘slippage’ mutations which arise in regions of the genome carrying simple repetitive sequences. The chances of creating mutations during DNA replication are not evenly distributed, and there are ‘hot spots’ for mutations, usually at hard-to-replicate regions [10]. One sequence characteristic that significantly increases mutation frequency is repetitiveness [11]. Repetitive regions tend to be unstable and play a role in various human diseases [12]. 

In this work, we utilized PCNA mutants that tend to randomly disassemble and fall from the chromatin in order to better understand the role of PCNA in preventing slippage mutations. Our results indicate that during replication, PCNA protects the genome from slippage mutations by physically encompassing DNA and preventing the denaturation of the two DNA strands, thus limiting events of misalignments of the two strands that can result in mutation. The stability of the PCNA ring on the chromatin is strengthened by its interaction with other proteins, and is a major determinant of its ability to reduce mutations at simple repeats. The genetic instability of repetitive sequences has a role in human diseases, and thus understanding the mechanisms responsible for such events is of major medical importance. 

## 2. Results and Discussion

**Figure 1:**
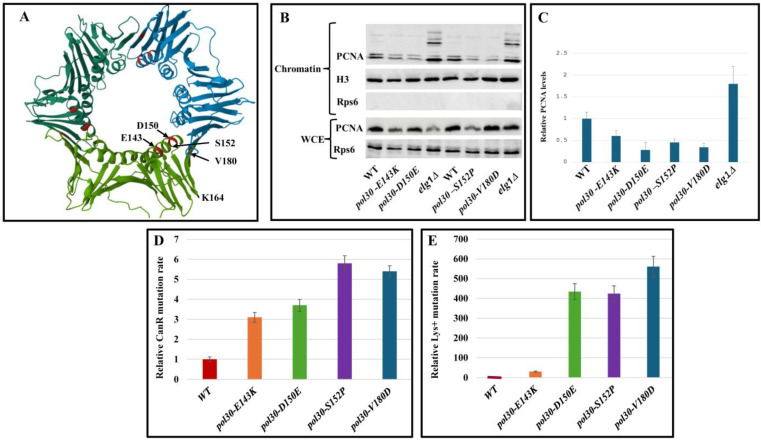
(**A**) Model of PCNA with the mutations marked on it. PDB accession number 1PLQ. (**B**) Western blot of the chromatin fraction showing the PCNA levels on the chromatin of a WT strain, the four PCNA alleles and an *elg1Δ* strain. (**C**) Quantification of PCNA levels on the chromatin from the Western blot shown at (**B**) and two more biological repeats. All DPP mutations have significantly (*p* value < 0.05) decreased amounts of PCNA on the chromatin compared to WT. Error bars represent standard deviation (SD) from the mean. (**D**) Mutation rates in the CAN forward mutation assay. WT and disassembly-prone *pol30* mutants shown. Error bars represent standard deviation (SD) from the mean. (**E**) Mutation rate as measured using a LYS assay. WT and disassembly-prone Pol30 mutants shown. Error bars represent standard deviation (SD) from the mean.

PCNA is a homotrimer sliding clamp complex essential for life. We sought to better understand what affects the stability of this complex and how this in turn affects DNA replication fidelity. In order to do so, we utilized four *POL30* alleles that carry mutations resulting in a labile homotrimer that tends to randomly disassociate from the chromatin (hereafter referred to as disassembly-prone PCNA or DPP alleles) [9]. These mutations were first characterized as showing ‘trimer interference’ [9], implying that they decrease the affinity between the subunits in the complex, leading to reduced stability. A model of yeast PCNA [13], with the location of the four mutations marked in red throughout the entire complex and specifically identified in one of the subunits, is shown in Figure 1A. We first validated the results found in the literature and measured the amount of PCNA retained on the chromatin in strains carrying these mutations (Figure 1B,C). Our results strengthen the previously published experiments [9,14], showing that all four mutants have decreased amounts of PCNA on the chromatin compared to the WT, and that *pol30-E143K* has the least severe defect. As a control, we show that cells lacking the *ELG1* gene, which is in charge of unloading PCNA from the DNA [15,16], has higher PCNA levels on the chromatin than wild type cells (Figure 1B,C). To estimate the effect of an unstable PCNA complex on DNA fidelity, we measured the mutation rate in strains carrying these *POL30* mutations. We calculated both forward mutation, using the CAN assay, and slippage mutation, using the *lys2-14A* allele (see Section 3, Methods). The first method measures all types of mutations that inactivate *CAN1*, resulting in resistance to the arginine analog canavanine, whereas the latter quantifies events of nucleotide insertion or deletion during DNA replication in a stretch of 14 A nucleotides that was introduced into the *LYS2* gene (hereafter referred to as slippage). In these assays, the DPP mutants exhibited elevated forward mutation rates and, in particular, a very high slippage mutation rate (Figure 1D,E). 

**Figure 2:**
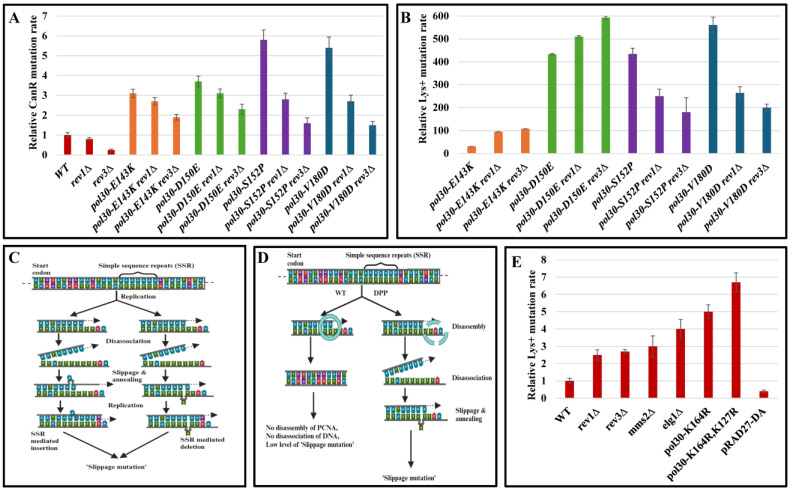
(**A**) Mutation rate in the CAN forward assay. WT and disassembly-prone Pol30 mutants shown with and without *rev1Δ* and *rev3Δ* mutations. Error bars represent standard deviation (SD) from the mean. (**B**) Mutation rate as measured using the LYS assay. WT and disassembly-prone *pol30* mutants shown with and without the *rev1Δ* and *rev3Δ* mutations. Error bars represent standard deviation (SD) from the mean. (**C**) An illustration of how deletion and insertion can occur when replicating a repetitive sequence. Random denaturing between DNA strands can result in the mis-annealing of the DNA that can either result in insertion or deletion. (**D**) How disassembly-prone PCNA alleles can affect slippage mutation rate. PCNA, encompassing the dsDNA, can physically prevent disassociation between the DNA templates, thus preventing repetitive sequence mis-annealing and subsequent mutations. (**E**) Mutation rate as measured using the LYS assay. WT is shown with and without *rev1Δ*, *rev3Δ*, *mms2Δ*, *elg1Δ*, *pol30-K164*, *pol30-K164R*,*K127R* and an overexpressing plasmid carrying Rad27-DA. Error bars represent standard deviation (SD) from the mean.

The error-prone DNA damage bypass pathway, executed by Polζ, is responsible for almost two thirds of the spontaneous mutations in yeast [17]. It is possible that the instability of PCNA leads to increased dependency on this pathway or to an increased occurrence of DNA damage, part of which is then handled by the error-prone pathway. We therefore measured the mutation rate of strains carrying the DPP alleles combined with *rev3Δ*, lacking the catalytic subunit of Polζ, or with *rev1Δ*, lacking a DNA polymerase that also functions as a regulatory accessory subunit for Polζ [18,19,20]. Deleting either gene decreased the rate of canavanine-resistant mutants in the WT background (Figure 2A). On the other hand, the effect of TLS inactivity on the slippage mutation rate was more complicated and unforeseen. First, *rev3Δ* or *rev1Δ* in a *pol30-E143K* or *pol30-D150E* background resulted in an **increase** in the mutation rate (Figure 2B), and second, even in the other two DPP mutants, where deleting *REV1* or *REV3* decreased mutation rate, it did not eliminate most of the mutations, meaning that in strains carrying these alleles, most slippage mutations are not caused by the activity of the error-prone polymerases, which agrees with previous findings [21].

Why does the deletion of error-prone DNA damage bypass genes increase the slippage mutation rate in the background of two of the DPPs while having the opposite phenotype in the two others? To answer this, we first needed to understand the source of slippage mutations, as measured in the LYS assay. One of the most established models for replication-associated slippage mutations [22] posits that they are created during DNA replication as a consequence of mis-alignment between the template and the replicated DNA strand in rows of identical nucleotides or short repeats (illustrated in Figure 2C,D). PCNA plays an important role in ensuring the processivity of the replicative DNA polymerase, and in addition, it encircles both strands of the DNA, restraining local separation between the DNA strands. Thus, DPP mutants, which tend to fall off the DNA, may facilitate mis-alignment events, which, in the 14 A insertion in *LYS2*, can result in slippage mutations. We hypothesize that deleting the error-prone bypass genes influenced the slippage rate not because of their biochemical activity, but rather because of their effect on the stability of the PCNA complex. Indeed, even single *rev1Δ* and *rev3Δ* mutations showed an increase in slippage mutations (Figure 2E). But why do different *pol30* alleles show opposite phenotypes when the error-prone polymerase is absent? 

**Figure 3:**
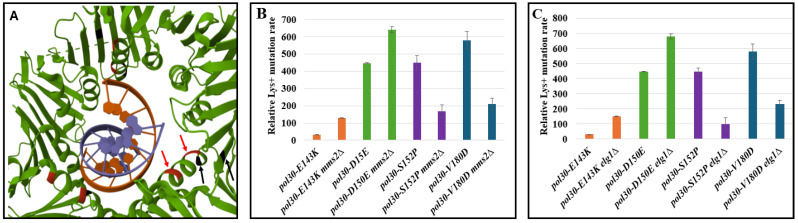
(**A**) Model of a PCNA mounted on DNA with the mutations marked on it. PDB accession number 3K4X. Residues E143 and D150 are in close proximity to the DNA (marked in red) in the innermost ring of PCNA while S152 and V180 are facing outward and away from the DNA (marked in black). (**B**) Mutation rate as measured using the LYS assay. WT and disassembly-prone *pol30* mutants shown with and without the *mms2Δ* mutation. Error bars represent standard deviation (SD) from the mean. (**C**) Mutation rate as measured using the LYS assay. WT and disassembly-prone Pol30 mutants shown with and without *elg1Δ*.

When they were originally isolated, all four mutants were referred to as ‘trimer interference mutants’ [9]. However, upon a closer look at the three-dimensional structure of PCNA encircling DNA (crystal structure [23], Figure 3A), mutations E143K and D150E (marked in red) are located at the innermost part of the ring, facing the DNA backbone and positioned less than 15Å from it. In contrast, the mutations S152P and V180D (marked in black) are oriented outward and situated more than 15Å away from the DNA backbone. This suggests that changing E143 and D150 may decrease the affinity of the complex to the DNA, thus reducing its stability. In this case, further interactions of PCNA with additional proteins such as Rev1 or Rev3 may help stabilize the labile complex. In addition to their outward orientation, residues S152 and V180 are in the vicinity of lysine 164, the target for ubiquitin and the SUMO modification of PCNA, which regulates the interaction with many proteins [24,25,26,27]. Such a proximity to additional interacting proteins may be significant: These mutations may decrease the stability of the complex by having lower affinity to some or all of the PCNA-interacting proteins. Thus, deleting a PCNA-interacting protein (such as a trans-lesion synthesis polymerase) will increase the complex stability and decrease the rate of slippage mutation.

**Figure 4:**
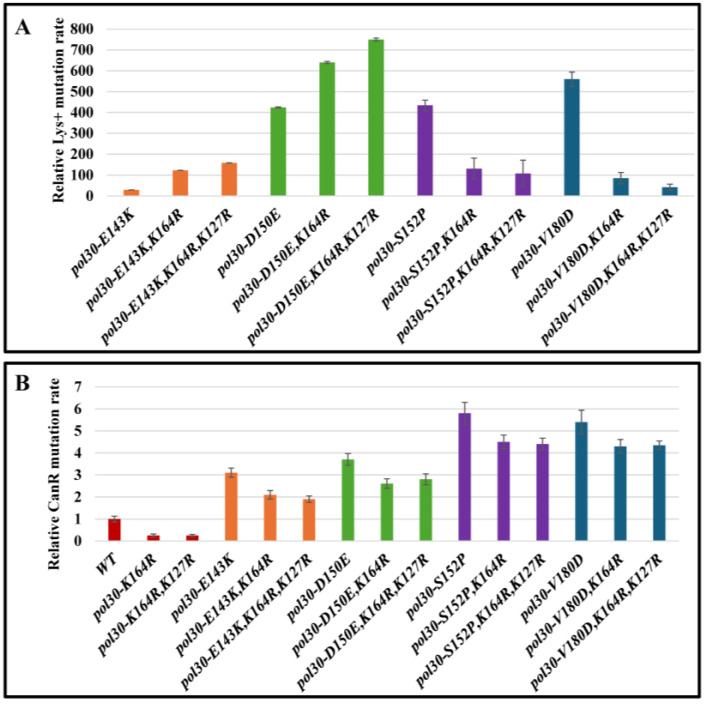
(**A**) Mutation rate as measured using the LYS assay. WT and disassembly-prone *pol30* mutants shown with and without *pol30-K164R* or *pol30-K164R*,*K127R*. Error bars represent standard deviation (SD) from the mean. (**B**) Mutation rate in the CAN forward assay. WT and disassembly-prone Pol30 mutants shown with and without *pol30-K164R* or *pol30-K164R*,*K127R*. Error bars represent standard deviation (SD) from the mean.

To test this hypothesis, we checked how the rate of slippage mutation is affected by deleting two additional genes that encode PCNA interacting proteins, Mms2 and Elg1 (Figure 3B,C). Mms2 forms a complex with Ubc13 and Rad5 to poly-ubiquinate PCNA [24,28]. Elg1 forms an RFC-like complex that unloads PCNA and is important for recycling PCNA during the S phase [8,15]. Thus, the two proteins play very different biochemical roles, but share the property of binding to PCNA. As expected from our hypothesis, the deletions differently affected the slippage mutation rate of the mutants similarly to the trend observed with *rev3Δ* and *rev1Δ*, even though they have different and distinct roles, which are also different from those of Rev3 and Rev1. This result strengthens the notion that what influences the slippage mutation rate is the physical interaction of these proteins with PCNA, and not their function. To further validate this, we combined the DPP alleles with a mutation of lysine 164 of Pol30. The ubiquitination of this residue controls the DDT pathways [29] and as such, mutating it will decrease the amount of proteins interacting with PCNA. In accordance with our hypothesis, Figure 4A shows that mutating lysine 164 to arginine increases the slippage mutation rate of *E143K* and *D150E* alleles while decreasing it in the background of *S152P* and *V180D* mutations, the same trend observed until now. Further mutating lysine 127, the second residue that can undergo SUMOylation, affected the slippage mutation rate even more (Figure 4A). This trend was observed only when measuring the rate of slippage mutation. When measuring forward mutation in the CAN assay, changing K164 or K164 and K127 together decreased the mutation rate, due to the inactivation of the DDT pathway (Figure 4B). 

**Figure 5:**
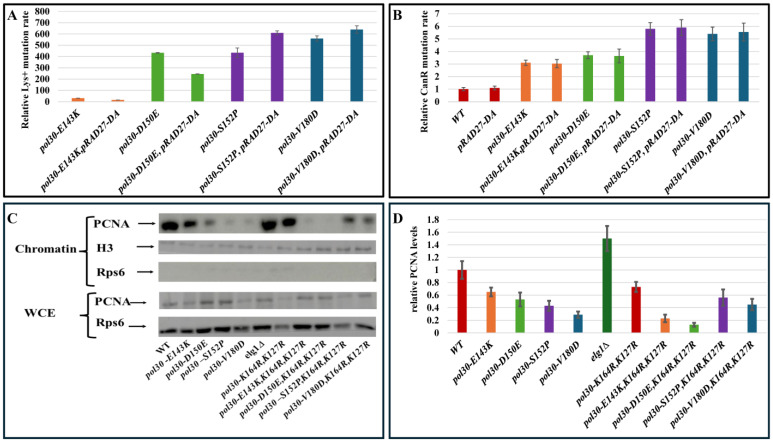
(**A**) Mutation rate as measured using the LYS assay. WT and disassembly-prone Pol30 mutants shown with and without an overexpressing plasmid carrying *rad27-DA*. Error bars represent standard deviation (SD) from the mean. (**B**) Mutation rate in the CAN forward assay. WT and disassembly-prone *pol30* mutants shown with and without an overexpressing plasmid carrying *rad27-DA*. Error bars represent standard deviation (SD) from the mean. (**C**) Western blot of the chromatin fraction showing the PCNA levels on the chromatin of a WT strain and the four PCNA alleles with and without *pol30-K164R*,*K127R* and an *elg1Δ* strain. (**D**) Quantification of this blot (and two more biological repeats) is shown. Error bars represent standard deviation (SD) from the mean.

To further support our explanation for the different effect of mutating PCNA-interacting proteins in different DPP mutants, we wanted to overexpress a protein that binds PCNA but has no biological role. Such a binding should affect the slippage mutation rates of the DPP mutants, even though the protein has no intrinsic activity. We overexpressed *rad27-DA* [30], a *RAD27* mutant that lacks any activity but is still able to interact normally with PCNA. Rad27 is an exonuclease that acts in Okazaki fragment maturation and in the process physically interacts with PCNA [31,32]. We overexpressed *rad27-DA* using a high-copy number plasmid in strains carrying a WT *RAD27* to avoid problems in Okazaki fragment maturation (no dominant negative effects are observed in cells overexpressing this allele). Overexpressing *rad27-DA* decreased the slippage mutation rate of the alleles *E143K* and *D150E* while increasing the slippage rate of strains with *S152P* and *V180D* (Figure 5A). This again validates our model, as the same mutants that showed an increased mutation rate in the absence of PCNA-interacting proteins showed a decrease in mutagenesis when *rad27-DA* was overexpressed, and vice versa. The effect of overexpressing *rad27-DA* was observed only when measuring the slippage mutation rate, and no significant effect was found in the CAN forward assay (Figure 5B). 

To provide additional confirmation to our model, we measured PCNA abundancy on the chromatin using the previously described fractionation protocol [33]. In Figure 5C,D, it is evident that lowering PCNA interactions by mutating either lysine 164 or both lysines 164 and 127 to arginine (and thus preventing the interaction of several proteins with ubiquitin- or SUMO- modified PCNA) lowers the amount of PCNA on the chromatin in *E143K* and *D150E* alleles while increasing it in the background of the *S152P* and *V180D* mutations. 

What do our results tell us about the function of PCNA under normal circumstances? The analysis of the DPP mutants teaches us that PCNA stability as a complex is increased by its interactions with other proteins, first, because deleting PCNA-interacting partners or mutating lysine 164 and 127 elevates the slippage mutation rate in a WT background (Figure 2E), and second, because overexpressing *rad27-DA* on a WT background decreases the slippage mutation rate (Figure 2E). Lastly, the strongest evidence is the fact that Pol30-K127R, K164R, which has no ubiquitination or SUMOylation, and thus cannot interact with most normally interacting proteins, has a lower amount of PCNA on the chromatin than a WT PCNA (Figure 5C,D). This means that during replication, the interaction of PCNA with other proteins increases the stability of the complex, possibly because they interact with several of the subunits at once, thus tightening all of the complex together. 

PCNA stability thus plays an important role in safeguarding the fidelity of DNA replication. This is particularly true for regions of the genome that contain simple repetitive sequences, which have a higher tendency to mis-align during replication. Indeed, mutations in repetitive sequences are common in several human diseases and in cancer.

## 3. Materials and Methods

### 3.1. Yeast Strains

A list of yeast strains is provided in Table 1.

### 3.2. Plasmids

A list of plasmids used is provided in Table 2.

### 3.3. Growth Media

YEAST: YPD (yeast rich medium)—1% Bacto yeast extract, 2% Bacto peptone, 2% Glucose. For solid media, 20 g/L agar was added.

SD (yeast defined medium)—0.67% Bacto yeast nitrogen base w/o amino acids, 2% Glucose. Amino acids were added according to requirement. For solid media, 20 g/L agar was added.

BACTERIA: LB—2% Bacto LB extract. Ampicillin (Amersham, Piscataway, NJ, USA) 50 mg/L was added to LB + Amp plates. For solid media, 20 g/L agar was added.

### 3.4. Bacteria and Plasmid Extraction

Plasmid pRS325-*rad27-DA* was extracted from the lab frozen stock (in *E. coli* DH5α cells), grown in LB and extracted using a Macherey Nagel (Duren, Germany) plasmid extraction kit.

### 3.5. Determining Yeast Spontaneous Mutation Rate

In order to determine the spontaneous mutation rate, we carried out fluctuation rate experiments as described in [36]. The first step was to plate a YPD plate with diluted yeast such that around 100 colonies per plate would grow. After exactly three days, 12 colonies were picked up, each to a different Eppendorf tube containing sterile water. Cells from each such tube were plated on three plates: (1) CAN plates, containing canavanine, measuring any mutations that would disturb the proper function of the *CAN1* gene; (2) SD-LYS plates, measuring the reversion of a 14-adenine insertion at the *LYS2* gene, which causes a frame shift; and (3) YPD after appropriate dilution to obtain a count of live cells. After three days, colonies were counted on all plates and mutation rates were calculated using the median Lea–Coulson 1949 method [37]. The mutation rates shown in figures are an average of three fluctuation test experiments normalized to WT.

### 3.6. Chromatin Fractionation Assay

A total of 50 mL of a logarithmic culture was collected and washed with SB (1 M Sorbitol, 20 mM Tris-HCl pH 7.4). Next, cells were suspended in 1 mL SB, 30 μL Zymolase 20 T (20 mg/mL in SB) was added, and samples were incubated at 30 °C until spheroplasts were visible (around 1 h). Spheroplasts were washed twice with SB and suspended in 500 mL EBX (20 mM Tris-HCl pH 7.4, 100 mM NaCl, 0.25% Triton X-100, 15 mM β-ME + protease/phosphatase inhibitors). Triton X-100 was added to 0.5% final to lyse the outer cell membrane, and the samples were kept on ice for 10 min with gentle mixing. Whole cell lysate (WCE) samples were taken and the rest of the lysate was layered over 1 mL NIB (20 mM Tris-HCl pH 7.4, 100 mM NaCl, 1.2 M Sucrose, 15 mM β-ME + protease/phosphatase inhibitors). After centrifugation (15 min in 12K RFC at 4 °C), the cytoplasmic fraction was taken. The nuclear pellet was suspended in 500 μL EBX and Triton X-100 added to 1% final to lyse the nuclear membrane. The pellet was centrifuged (10 min 15K RFC at 4 °C) and the chromatin was suspended in 50 μL Tris pH 8.0 for Western blot analysis (Chromatin).

### 3.7. Western Blot Analysis

Proteins extracted from the fractionation protocol, either from the chromatin fraction or the WCE, were loaded on an acrylamide gel prepared in our lab in 15% acrylamide concentration for PCNA, RPS6 and H3 and on 8% for Srs2 and Rev1 (HA tagged). Gels were run for 90 min in 140 V in a Bio-Rad Western blot apparatus (Hercules, CA, USA). Afterwards, the gel was taken apart and proteins were transferred using the Bio-Rad transfer apparatus for 90 min in 400 mA to cellulose membranes. The membranes were incubated for 30 min in 1% skim milk for blocking and afterwards incubated with primary antibody overnight (anti-PCNA: Sc65598-Santa Cruz, Dallas, TX, USA; anti-Flag: F1804-Sigma-Aldrich, St. Louis, MO, USA; anti-H3: ab1791-abcam, Cambridge, UK; anti-Srs2: sc11991-Santa Cruz, Dallas, TX, USA; anti-RPS6: ab40820-abcam, Cambridge, UK, anti-HA:SC-8036 Santa Cruz, Dallas, TX, USA). On the following morning, the membranes were washed 5 times in TTBS (tris-buffered saline), incubated after each wash for 10 min leading to an hour incubation in a secondary antibody and then there was another cycle of 5 washes. In the end, using Thermo Scientific ECL (Waltham, MA, USA), we exposed the membranes in imager600 and captured pictures of the membranes with different exposures.

## Figures and Tables

**Table 1:** Yeast strain list.

Name	Relevant Genotype	Source and Number in Lab Stock
E134	*MAT*α *ade5-1 lys2::InsE-A14 his7-2 leu2-3*,*112 trp1-289 ura3-52*	[34]
E134 *pol30-S152P*	*Same*, *POL30-S152P:LEU2*	This work 17,821
E134 *pol30-V180D*	*Same*, *pol30-V180D:LEU2*	This work 17,822
E134 *pol30*-*E143K*	*Same*, *pol30-E143K:LEU2*	This work 17,823
E134 *pol30*-*D150E*	*Same*, *pol30-D150E:LEU2*	This work 17,824
E134 *pol30*-*S152P* *mms2∆*	*Same*, *pol30-S15SP:LEU2*, *mms2::KanMX*	This work 17,825
E134 *pol30*-*S152P* *rev1∆*	*Same*, *POL30-S152P:LEU2*, *rev1::URA3*	This work 17,826
E134 *pol30*-*S152P* *rev3∆*	*Same*, *pol30-S152P:LEU2*, *rev3::KanMX*	This work 18,081
E134 *pol30*-*S152P* *elg1∆*	*Same*, *pol30-S152P:LEU2*, *elg1::HYG*	This work 18,758
E134 *pol30*-*S152P* *rev1∆*, *rev3∆*	*Same*, *pol30-S152P:LEU2*, *rev1::URA3*, *rev3::KanMX*	This work 19,204
E134 *POL30-V180D* *mms2∆*	*Same*, *pol30-V180D:LEU2*, *mms2::KanMX*	This work 17,827
E134 *POL30-V180D* *rev1∆*	*Same*, *pol3030-V180D:LEU2*, *rev1::URA3*	This work 17,831
E134 *POL30-V180D* *rev3∆*	*Same*, *pol30-V1D80:LEU2*, *rev3::KanMX*	This work 18,082
E134 *pol30-V180D* *elg1∆*	*Same*, *pol30-V180D:LEU2*, *elg1::HygMX*	This work 18,065
E134 *POL30-V180D* *rev1∆*, *rev3∆*	*Same*, *pol3030-V180D:LEU2*, *rev1::URA3*, *rev3::KanMX*	This work 19,205
E134 *POL30-E143K* *mms2∆*	*Same*, *pol3030-E143K:LEU2*, *mms2::KanMX*	This work 18,367
E134 *pol30-E143K* *rev1∆*	*Same*, *pol30-E143K:LEU2*, *rev1::URA3*	This work 17,829
E134 *pol30-E143K* *rev3∆*	*Same*, *pol30-E143K:LEU2*, *rev3::KanMX*	This work 18,368
E134 *pol30-E143K* *elg1∆*	*Same*, *pol30-E143K:LEU2*, *Elg1::HygMX*	This work 18,052
E134 *POL30-E143K* *rev1∆*, *rev3∆*	*Same*, *pol30-E143K:LEU2*, *rev1::URA3*, *rev3::KanMX*	This work 18,366
E134 *pol30*-*D150E* *mms2∆*	*Same*, *POL30-D150E:LEU2*, *mms2::KanMX*	This work 18,912
E134 *pol30*-*D150E* *rev1∆*	*Same*, *pol30-D150E:LEU2*, *rev1::URA3*	This work 17,828
E134 *pol30*-*D150E* *rev3∆*	*Same*, *pol30-D150E:LEU2*, *rev3::KanMX*	This work 18,083
E134 *pol30*-*D150E* *elg1∆*	*Same*, *pol30-D150E:LEU2*, *elg1::HygMX*	This work 17,830
E134 *pol30*-*D150E* *rev1∆*, *rev3∆*	*Same*, *pol30-D150E:LEU2*, *rev1::URA3*, *rev3::KanMX*	This work 19,203
E134 *pol30-S152P*,*K164R*	*Same*, *pol30-S152P*, *K164R:LEU2*	This work 19,684
E134 *pol30-V180D*,*K164R*	*Same*, *pol30-V180D*, *K164R:LEU2*	This work 19,311
E134 *pol30-E143K*,*K164R*	*Same*, *pol30-E143K*, *K164R:LEU2*	This work 19,525
E134 *pol30-D150E*,*K164R*	*Same*, *pol30-D150E*, *K164R*, *K127R:LEU2*	This work 19,604
E134 *pol30-S152P*,*K164R*,*K127R*	*Same*, *pol30-S152P*, *K164R*, *K127R:LEU2*	This work 19,685
E134 *pol*30*-V180D*,*K164R*,*K127R*	*Same*, *pol30-V180D*, *K164R*, *K127R:LEU2*	This work 19,678
E134 *pol30-E143K*,*K164R*,*K127R*	*Same*, *pol30-E143K*, *K164R*, *K127R:LEU2*	This work 19,527
E134 *pol*30-*D150E*,*K164R*,*K127R*	*Same*, *pol30-D150E*, *K164R*, *K127R:LEU2*	This work 19,604
E134 *pol*30-*K164R*,*K127R*	*Same*, *pol30-K164R:LEU2*	This work 19,809
E134 *pol*30-*K164R*	*Same*, *pol30-K164R: LEU2*	This work 19,526

**Table 2:** Plasmid list.

pRS325-*rad27-DA*	*rad27-DA* with ADH1 promoter in pRS325 (*LEU2* Marker, 2micron)	[35] 3559

## Data Availability

The original contributions presented in the study are included in the article, further inquiries can be directed to the corresponding author/s.
